# A Deep Learning Approach for Neuronal Cell Body Segmentation in Neurons Expressing GCaMP Using a Swin Transformer

**DOI:** 10.1523/ENEURO.0148-23.2023

**Published:** 2023-09-25

**Authors:** Mohammad Shafkat Islam, Pratyush Suryavanshi, Samuel M. Baule, Joseph Glykys, Stephen Baek

**Affiliations:** 1 School of Data Science, The University of Virginia, Charlottesville, VA 22903; 2 Department of Pediatrics, Iowa Neuroscience Institute, The University of Iowa, Iowa City, IA 52242; 3 Department of Biomedical Engineering, The University of Iowa, Iowa City, IA 52242; 4 Department of Neurology, The University of Iowa, Iowa City, IA 52242

**Keywords:** convolutional neural networks, fluorescent, GCaMP, neuron, segmentation, vision transformers

## Abstract

Neuronal cell body analysis is crucial for quantifying changes in neuronal sizes under different physiological and pathologic conditions. Neuronal cell body detection and segmentation mainly rely on manual or pseudo-manual annotations. Manual annotation of neuronal boundaries is time-consuming, requires human expertise, and has intra/interobserver variances. Also, determining where the neuron’s cell body ends and where the axons and dendrites begin is taxing. We developed a deep-learning-based approach that uses a state-of-the-art shifted windows (Swin) transformer for automated, reproducible, fast, and unbiased 2D detection and segmentation of neuronal somas imaged in mouse acute brain slices by multiphoton microscopy. We tested our Swin algorithm during different experimental conditions of low and high signal fluorescence. Our algorithm achieved a mean Dice score of 0.91, a precision of 0.83, and a recall of 0.86. Compared with two different convolutional neural networks, the Swin transformer outperformed them in detecting the cell boundaries of GCamP6s expressing neurons. Thus, our Swin transform algorithm can assist in the fast and accurate segmentation of fluorescently labeled neuronal cell bodies in thick acute brain slices. Using our flexible algorithm, researchers can better study the fluctuations in neuronal soma size during physiological and pathologic conditions.

## Significance Statement

Neuronal cell body partitioning is essential for evaluating the effects of physiological and pathologic conditions. Neuronal segmentation is challenging because of the complex morphologic structures of neuronal cell bodies and their surroundings. Most current approaches for detecting and segmenting neurons are based on manual or pseudo-manual annotations of the neuronal boundaries by human experts. These are time-consuming and have intra/interobserver variability. Leveraging the current success of vision transformers for general object detection and segmentation tasks, we developed a deep-learning-based approach for automated, fast, robust 2D neuronal cell body segmentation using a state-of-the-art vision transformer (Swin transformer). This approach for neuronal cell body segmentation can assist researchers in evaluating the changes in neuronal cell body sizes under different pathologic conditions.

## Introduction

The accurate estimation of cellular morphology is essential for identifying cytotoxic edema of neurons and astrocytes in multiple pathologic conditions, including brain injury, seizures, hypoxia, and ischemia (Andrew et al., 2007; Risher et al., 2009; Glykys et al., 2017). Despite these critical needs, measuring neuronal cell bodies remains challenging. Neurons have complex structures with multiple branches, and calculating the changes in somatic and dendritic sizes is arduous. Most common detection methods involve manual or semi-automatic measurement of the neuronal area, which can overlook small but relevant morphologic changes. This labor-intensive process also carries a high degree of human bias because of its manual nature. Moreover, it is challenging to determine when a neuronal body ends and when processes like dendrites and axons begin unless unique markers are used. Thus, there is an urgent need for automatic, reproducible, fast, and unbiased approaches to detect and segment neuronal cell bodies with high precision. The lack of such methods impedes the understanding of pathways that regulate neuronal size during various brain injuries.

Identifying neuronal cell bodies that express diverse biosensors in acute brain slices is taxing. The imaged tissue, commonly between 350 and 450 μm thick, has overlapping neurons across different depth planes, fluorescence intensity variations throughout the thickness of a brain slice, and low contrast between neuronal cell bodies and the neuropil, among others. As a result, current state-of-the-art deep-learning-based cell segmentation techniques (Stringer et al., 2021; Greenwald et al., 2022) cannot be directly applied to segment neuronal boundaries in thick brain slices. A mask region-based convolutional neural network (Mask R-CNN; Kirillov et al., 2019) system was recently developed, called ANMAF, which automatically outlines fluorescent neuronal somatic areas in acute brain slices (Tong et al., 2021). However, biosensors with dim or fluctuating fluorescence or slices with a high neuropil signal can hinder contrast-based detection of neuronal cell bodies, making ANMAF less efficient, probably because of its limited ability to build long-range dependencies and global image contexts.

Vision transformers (Carion et al., 2020; Dosovitskiy et al., 2020) were developed to address CNN’s limited ability to build long-range dependencies and global contexts in images, and they were inspired by transformers used in the natural language processing (NLP) domain (Vaswani et al., 2017). While these vision transformers can construct long-range dependencies and global contexts, their computational complexity is quadratic. Recently, Swin transformers have achieved state-of-the-art performance in object detection by using a hierarchical feature representation and a shifted window (therefore the acronym Swin) approach while maintaining a linear computational complexity (Lin et al., 2021).

Building on the success of vision transformers, we developed an automated, fast, accurate, reproducible, and unbiased neuronal cell body segmentation algorithm using 2D two-photon images by optimizing the current state-of-the-art Swin transformer (Lin et al., 2021). Our algorithm detects neurons genetically expressing GCaMP6s, a widely used Ca^2+^-sensitive fluorophore (Chen et al., 2013), and stable yellow fluorescent protein (YFP). It also segments neuronal cell bodies in neurons expressing GCaMP6s exposed to excitotoxic insult and with higher detection rates than two different CNN algorithms (Mask-R CNN-based algorithm: ANMAF, and a CNN-based approach: Cellpose). Thus, our Swin transformer will allow researchers to measure changes in fluorescent neuronal cell bodies during different pathologic conditions.

## Materials and Methods

### Experimental design

Acute brain slices were prepared from neonatal mice (postnatal days 8–12) expressing neuronal GCaMP6s (C57BL/6J-Tg (Thy1-GCaMP6s) GP4.3Dkim/J Strain #024275, The Jackson Laboratory), or yellow fluorescent protein (YFP; B6.Cg-Tg(Thy1-YFP)HJrs/J Strain #003782, The Jackson Laboratory). Mice of both sexes were anesthetized with inhaled isoflurane and decapitated per a protocol approved by The University of Iowa. The brain was removed and placed in ice-cold artificial CSF (aCSF) containing (in mm) NaCl (120), KCl (3.3), CaCl_2_ (1.3), MgCl_2_ (2), NaH_2_PO_4_ (1.25), NaHCO_3_ (25), and D-glucose (10) with pH 7.3–7.4 when bubbled with carbogen (95% O_2_ and 5% CO_2_). Coronal brain slices 450 μm thick were cut using a vibratome (Leica VT1000S) while submerged in aCSF containing 2 mm kynurenic acid to block glutamatergic receptors. The brain slices were placed in an interface holding chamber containing aCSF (1.3 mm MgCl_2_) at room temperature for 30 min, after which the temperature was slowly increased to and maintained at 30°C. Slices were stored for at least 1 h before being transferred to the recording chamber. NMDA was obtained from Sigma, and stock solutions were prepared and diluted to 30 μm in aCSF on the experimental day.

### Optical imaging and manual detection

**Figure 1.**
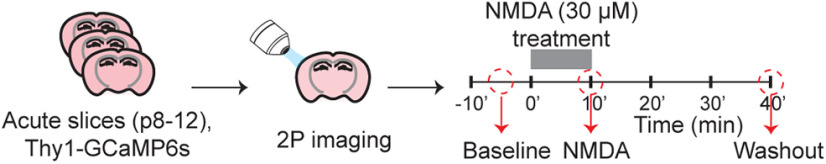
Experiment design. Experimental design and acquisition time-points of multiphoton Z-stacks for the three conditions: during baseline aCSF, NMDA perfusion, and washout. An identical approach was used to acquire images from Thy1-YFP-expressing neurons.

Two-photon laser scanning microscopy (2PLSM) was used to image neurons expressing genetically encoded fluorophores in layer IV/V of the somatosensory neocortex and CA1 hippocampal region. Acute brain slices were placed in a submerged chamber constantly perfused with aCSF maintained at 30°C. The location of the sensory neocortex was determined using epifluorescence. 2PLSM imaging was performed using the Bruker Ultima galvo-resonant system using an Olympus BX51WIF upright microscope body with a water immersion objective (20×, 1.0 N.A.). A Ti: sapphire tunable laser (Mai Tai HPDS; Spectra-Physics) generated two-photon excitation (920 nm: GCaMP6s; 860 nm: YFP). Scanning was performed with galvo-mirrors. Emitted light was bandpass filtered at 565 nm using a dichroic mirror (T510lpxrxt, Chroma), and green and yellow emission wavelengths were isolated using specific filters: 525/35 nm (green) and 535/30 nm (yellow). GaAsP or multialkali photomultiplier tubes (PMT, Hamamatsu Photonics) were used to acquire the emitted signal. Three-dimensional stacks (3D) of raster scans in the *xy* plane were imaged at 2-μm intervals with a 512 × 512-pixel resolution. All images were acquired at 2× digital zoom. All fluorophores were imaged at multiple depths. Ca^2+^ signals in GCaMP6s expressing neurons were imaged during baseline aCSF (low fluorescence), 30 μm NMDA perfusion (10 min, high fluorescence), and washout (up to 40 min, mid-high fluorescence; Fig. 1). A similar protocol was used for neurons expressing YFP. Images were background subtracted, smoothened (median filter, radius = 2), converted to maximum intensity projections (MIPs; every 10 images), and contrast-enhanced (CLAHE) using ImageJ. Thus, the maximal somatic areas for each neuron over 20 μm depth were represented in a MIP, allowing comparisons of the neuronal maximal areas between different experimental conditions (baseline, NMDA, and washout; Takezawa et al., 2023). For manual tracing, the contours of the neurons were generated using ImageJ and the Canny edge detector plugin.

### Deep neural network training

The MIPs of 20 μm depth were used to train and evaluate the performance of the deep neural network. To ensure proper separation of training/validation and testing datasets, the Swin transformer was assessed on the independent testing data, which was not used during training/validation. Thus, we first divided the total dataset into two sets: the training/validation dataset (D_1_, 75 images) and the testing dataset (D_2_, 15 images). Because of the labor-intensive manual annotation process (see below), generating exhaustive labels of all the neurons in the dataset is almost impossible. Hence, we generated a synthetic training/validation dataset (Syn_D1_) from D_1_ (∼700 manually annotated neurons), which was then used to train the neural network architecture, determine the hyperparameters, and optimize the model. Once the hyperparameters were determined and the network architecture was optimized, the approach was evaluated on the independent testing dataset (D_2_) and an independent synthetic testing dataset (Syn_D2_), which was never seen by the network before. The Swin transformer was trained on a Linux machine with a single GPU (NVIDIA RTX 5000). However, the approach can also be trained and tested on CPU-only machines. The CNN-based algorithms ANMAF and Cellpose were also trained using the same training/validation dataset (Syn_D1_), and their performances were evaluated on the same independent testing dataset (D_2_ and Syn_D2_).

### Manual tracings of somatic perimeter

To generate the synthetic dataset for training the deep neural network and to evaluate the performance of the Swin transformer network, two human experts with prior experience manually annotated a subset of prominent somatic regions from the training/validation dataset (D_1_, *n* = 75 Z-stack images, 6 brain slices) and the testing dataset (D_2_, *n* = 15 Z-stack images, 8 brain slices). Human experts proactively manipulated image intensity and contrast and examined adjacent slices to obtain contextual information to ensure the highest possible performance during manual contouring. These manual tracings were used as “reference standards” (ground truth) to train the deep neural network and quantitatively evaluate the performance of the Swin transformer approach.

### Quantitative evaluation of the algorithms

The Dice coefficient was used to measure the pixel-wise overlap between the predicted segmentation and the ground truth, as defined by the following equation:

$$Dice\;coefficient=\frac{2\left|X\;\cap^{}\;Y\right|}{\left|X\right|\;+\;\left|Y\right|}$$

We also used the intersection-over-union (IOU) parameter, also known as the Jaccard index, which is the area of overlap between the predicted segmentation and the ground truth divided by the area of union between the two.

$$IOU=\frac{\left|X\;\cap^{}\;Y\right|}{\left|X\;\cup^{}\;Y\right|}$$

The detected neurons were labeled either as true positives (TPs) if the instances were correct detections, false positive (FP) if the detection was incorrect (either background, wrong boundaries, or multiple instances being predicted as one neuron), or false negative (FN) if the approach missed the neuron. We also computed precision and recall according to the following equations:

$$Precision=\frac{True\;Positive\;(TP)}{True\;Positive\;(TP)+False\;Positive\;(FP)}$$

$$Recall=\frac{True\;Positive\;(TP)}{True\;Positive\;(TP)+False\;Negative\;(FN)}$$

The yield rate was defined by the ratio between the total number of neurons correctly detected by the approach in consideration and the total number of ground truth neurons.

### Statistical analysis

The normality of distributions was determined using the Shapiro–Wilk, Kolmogorov–Smirnov tests and Q-Q plots. Data are presented as mean ± confidence interval (95% CI). Repeated measure one-way ANOVA with the Dunnett test was used to compare multiple parametric data to a single group. Two-way ANOVA was used to study the interaction between algorithms and experimental intervention. Statistical significance was considered at *p* < 0.05 (Table 1).

**Table 1:** Statistical table

Figure	Graph	Structure	Type of test	Test result	
				*p*-value	*Post hoc*
Fig. 8	*B*	Normal	RM one-way ANOVA	*F*_(2,212)_ = 4.31, *p* = 0.015	Dunnett’s: *p* = 0.224 (Swin/ANMAF) Dunnett’s: *p* = 0.269 (Swin/Cellpose)
Fig. 8	*C*	Normal	RM one-way ANOVA	*F*_(2,212)_ = 4.57, *p* = 0.011	Dunnett’s: *p* = 0.209 (Swin/ANMAF) Dunnett’s: *p* = 0.248 (Swin/Cellpose)
Fig. 9	*B* (base)	Normal	RM one-way ANOVA	*F*_(2,48)_ = 8.72, *p* = 0.0006	Dunnett’s: *p* = 0.0004 (Swin/Manual) Dunnett’s: *p* = 0.5230 (Swin/ANMAF)
Fig. 9	*B* (NMDA)	Normal	RM one-way ANOVA	*F*_(2,82)_ = 8.695, *p* = 0.0004	Dunnett’s: *p* = 0.0004 (Swin/Manual) Dunnett’s: *p* = 0.6743 (Swin/ANMAF)
Fig. 9	*B* (wash)	Normal	RM one-way ANOVA	*F*_(2,56)_ = 7.840, *p* = 0.0010	Dunnett’s: *p* = 0.0011 (Swin/Manual) Dunnett’s: *p* = 0.8091 (Swin/ANMAF)
Fig. 9	*C*	Normal	Two-way ANOVA	Interaction *F*_(4,141)_ = 0.32, *p* = 0.86 Treatment *F*_(2,141)_ = 20.33, *p* < 0.0001 Detection algorithm *F*_(2,141)_ = 2.67, *p* = 0.073	
Fig. 9	*D*	Normal	Two-way ANOVA	Interaction *F*_(4,141)_ = 0.4295, *p* = 0.787 Treatment *F*_(2,141)_ = 22.88, *p* < 0.0001 Detection algorithm *F*_(2,141)_ = 1.381, *p* = 0.255	

### Code accessibility

The code described in the manuscript is freely available online at https://github.com/stephenbaek/cell-transformer. The code is available as Extended Data 1.

## Results

### Synthetic data generation

**Figure 2.**
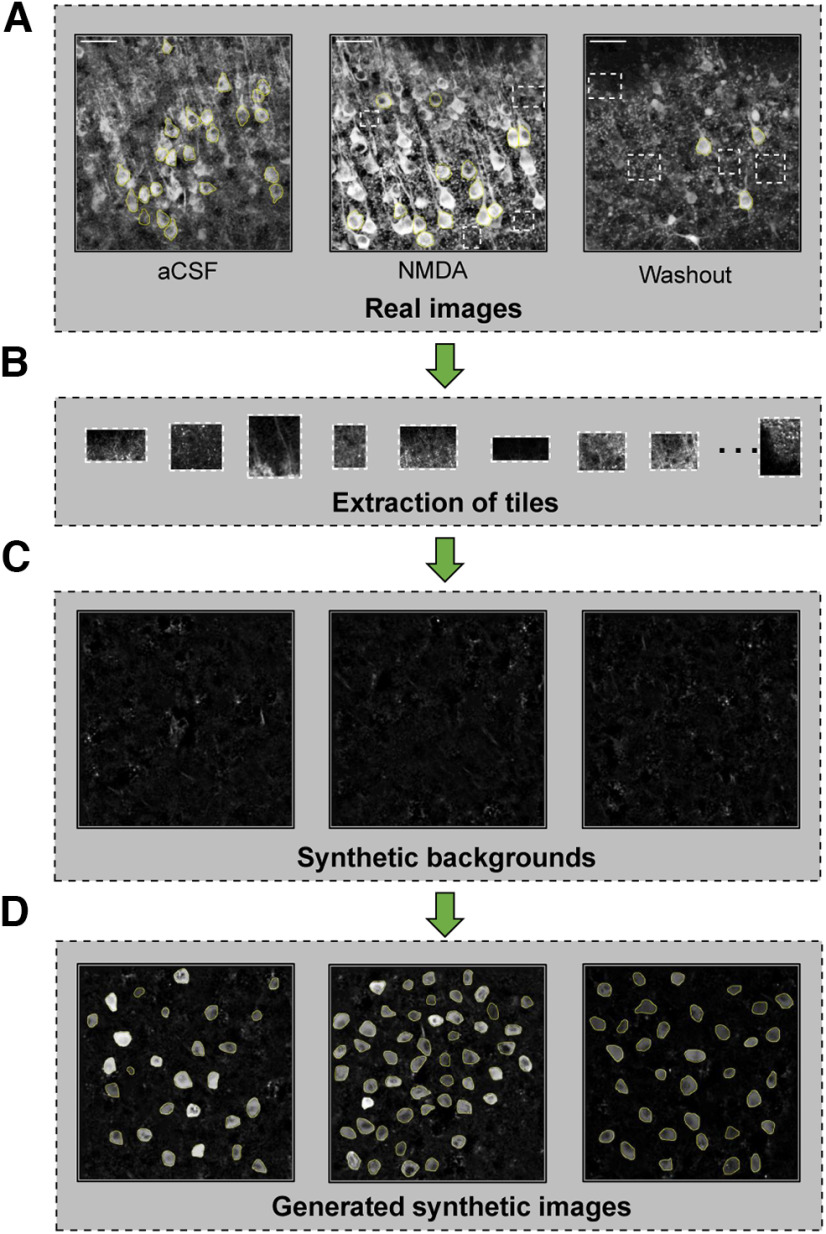
Generation of synthetic neuronal soma images. ***A***, Three examples of GCaMP6s-expressing neurons imaged under two-photon microscopy under three conditions: baseline aCSF, NMDA perfusion, and washout. The white rectangles represent the background, and the yellow outlines represent the soma boundaries traced by a human expert. ***B***, Background extraction. ***C***, Synthetic background generation from the extracted tiles. The background tiles were shuffled and randomly placed to generate the synthetic background. ***D***, Generation of synthetic neuronal images. The manually identified neuronal somatic boundaries were cut, pasted, and blended into the randomly selected background images to generate synthetic images. Scale bar = 50 μm.

Training a deep neural network requires an extensive collection of input images with manually annotated labels. However, exhaustive labeling of all instances of somatic boundaries on the neuronal images is highly time-consuming for a human expert. Hence, to train a Swin transformer, we first generated synthetic neurons (Fig. 2). A human expert manually annotated a few GCaMP6s-expressing neurons imaged in acute brain slices during baseline, NMDA perfusion and washout conditions (capturing different fluorescence intensities and somatic sizes) and nonsomatic regions from the input images to generate a collection of background tiles (*n* = 610 cells, 44 backgrounds; Fig. 2*A*,*B*). The background tiles were placed randomly and blended to avoid unnatural contrast between neighboring tiles to create a collection of synthetic background images (Fig. 2*C*). Finally, the manually identified somatic boundaries were pasted into randomly selected synthetic background images to produce a collection of synthetic cell images (Fig. 2*D*). From the training/validation dataset (D_1_, *n* = 75 Z-stack images, 6 brain slices) and the independent testing dataset (D_2_, *n* = 15 Z-stack images, 8 brain slices), we generated 3000 synthetic training/validation images (Syn_D1_) and 1000 synthetic separate testing images (Syn_D2_). We used Syn_D1_ to train the Swin transformer-based deep learning network.

### The deep neural network architecture

**Figure 3.**
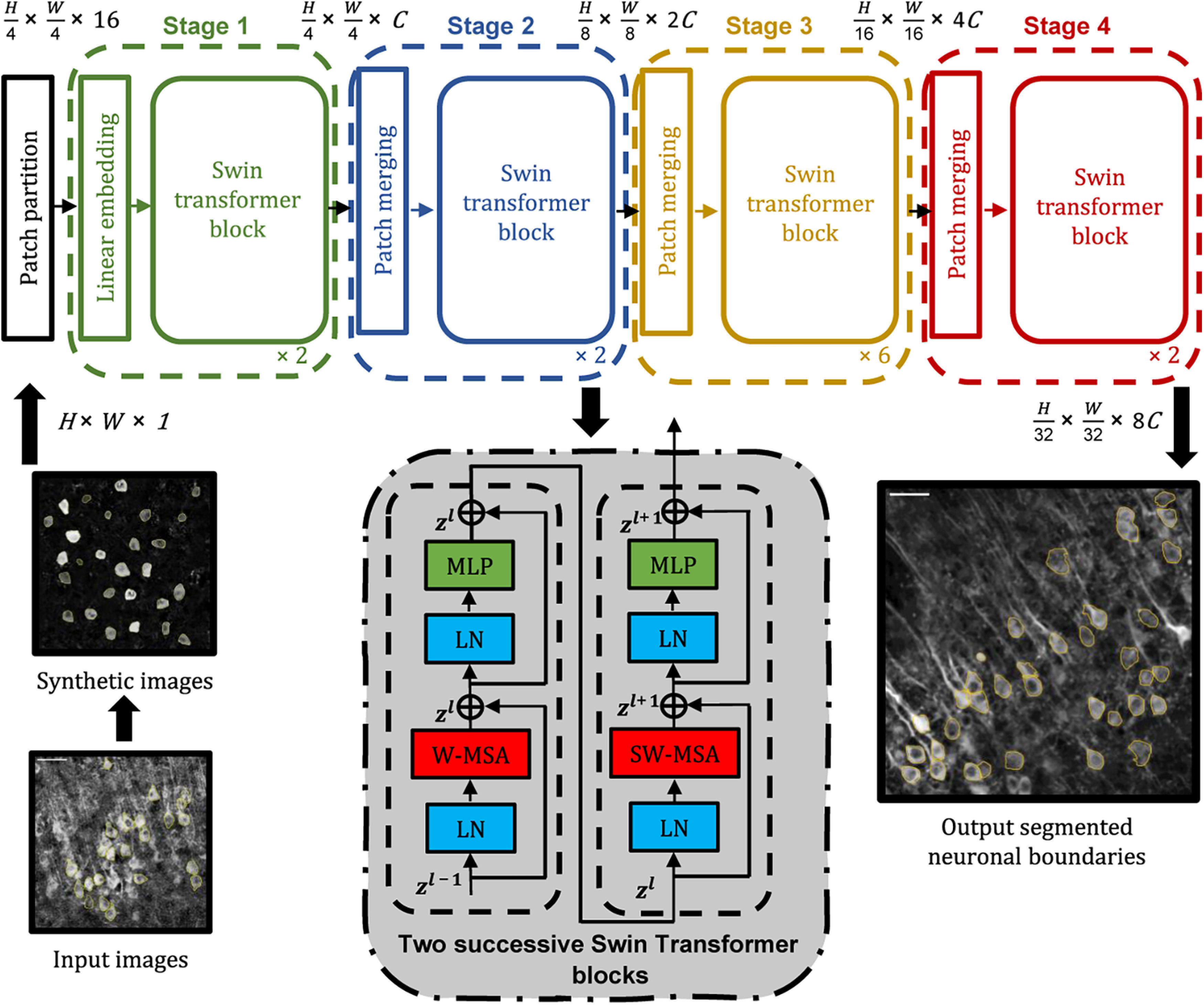
Deep learning network architecture based on the Swin transformer. Two successive Swin transformer blocks are shown at the bottom. W-MSA and SW-MSA represent multihead self-attention modules with regular and shifted windows. The orange outlines represent the output neuronal boundaries identified by the Swin transformer. Scale bar = 50 μm.

The architecture of the deep neural network based on the Swin transformer (Lin et al., 2021) is shown in Figure 3. The neural network consists of four stages with four components: patch partition module, linear embedding, Swin transformer block, and patch merging module. The patch partition module separates the grayscale input images into nonoverlapping 4 × 4 patches. In the NLP domain, tokenization is employed for cutting input text data into meaningful parts, called “tokens,” which can be embedded into a vector space and fed into the transformer. Like the NLP domain transformers, each patch is considered a token with a feature dimension of 4 × 4×1. Next, the linear embedding layer projects these features into an arbitrary dimension, *C *=* *96. This arbitrary dimension, *C*, determines the size of the Swin transformer. We used a small Swin transformer (*C *=* *96) for lower computational complexity. The patches are then processed by multiple Swin transformer blocks, which preserve the number of patch tokens. The Swin transformer block has a shifted window-based multihead self-attention module (SW-MSA) created by replacing the standard multihead self-attention (MSA; Dosovitskiy et al., 2020) with a shifted window-based module and a two-layer multilayer perceptron (MLP). The shifted window partitioning provides higher efficiency than standard multihead self-attention by computing features within local windows and allowing cross-window connections. Before each SW-MSA and MLP, LayerNorm is applied, and a residual connection is made after each module. LayerNorm is a simpler normalization method that transforms the inputs to have zero mean and unit variance for each batch across all elements. The linear embedding module, followed by two consecutive Swin transformer blocks, is Stage 1.

**Figure 4.**
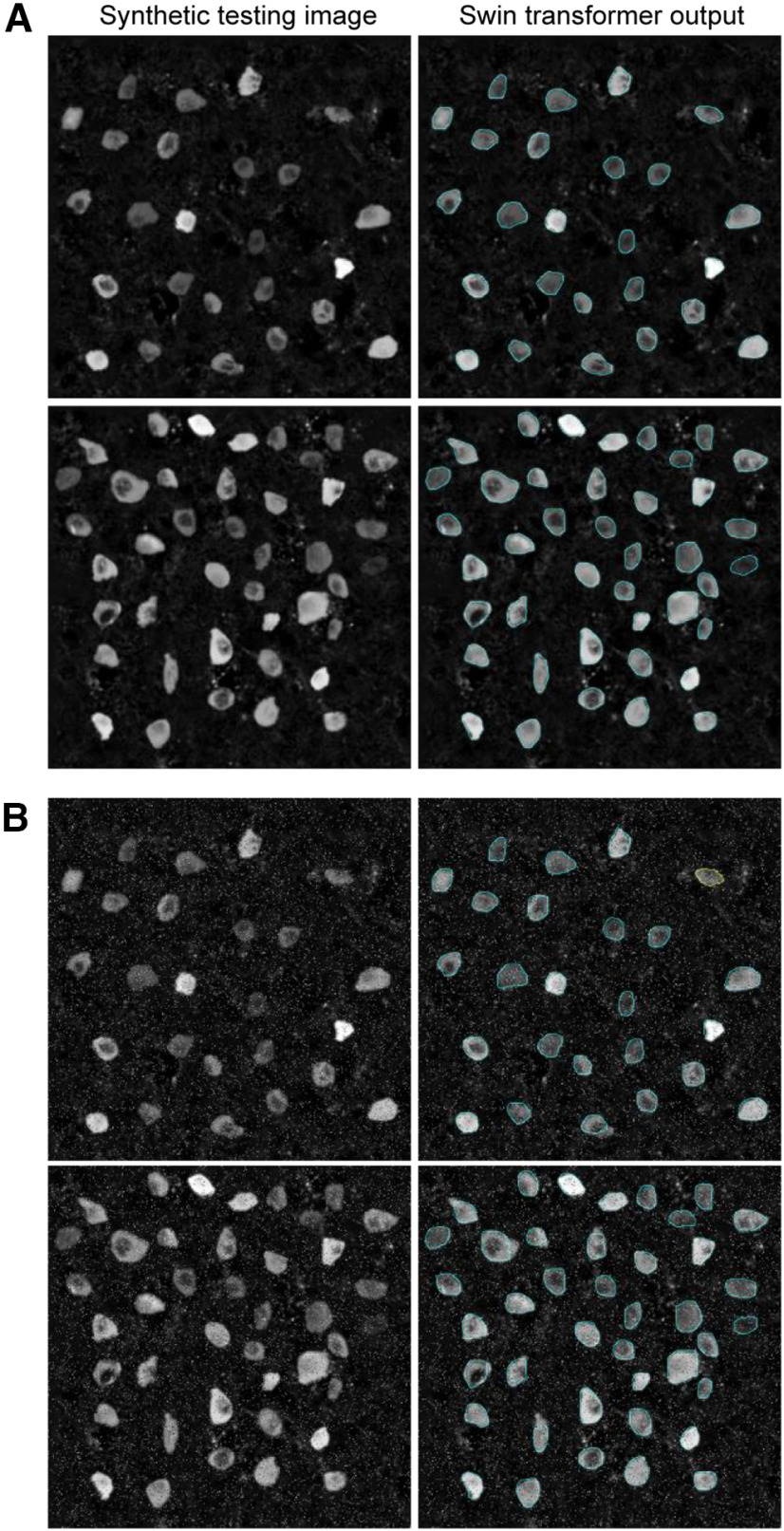
The Swin transformer detected neuronal boundaries in the independent testing dataset (Syn_D2_). ***A***, Left column, Synthetically generated images without noise. Right column, Swin transformer detected boundaries. ***B***, Same as ***A*** in the presence of salt and pepper noise. Cyan outlines: true positive detections by the Swin transformer. Yellow outlines: false negatives.

Stage 2 consists of a patch merging module and two consecutive Swin transformer blocks. The patch merging layer merges the features from each group of 2 × 2 neighboring patches and applies a linear layer on the 4*C* dimensional concatenated features. The output from the patch merging layer has a dimension of 2*C*, with fewer patch tokens. Since the patch merging layer merges features from neighboring 2 × 2 patches, the number of tokens is reduced by a multiple of 2 × 2 = 4, and the resolution is downsampled by a factor of 2, resulting in $\frac{H}{8}\times \frac{W}{8}$. Next, two consecutive Swin transformer blocks are applied, with resolutions of $\frac{H}{8}\times \frac{W}{8}$. Stages 3 and four consist of similar modules and keep the resolutions at $\frac{H}{\mathrm{16}}\times \frac{W}{\mathrm{16}}$ and $\frac{H}{\mathrm{32}}\times \frac{W}{\mathrm{32}}$. The denominators represent further downsampling of resolutions of 2 by the patch merging layer. These four stages produce a hierarchical representation. Once trained, this deep learning network generates the output segmentations of the neural cell bodies from the input images with the corresponding confidence scores for each instance. We used thresholding to remove objects with lower confidence scores. The synthetic training/validation dataset (Syn_D1_) was used for architecture design and training to determine the hyper-parameters of the Swin transformer. Using this architecture, the Swin transformer correctly identified neuronal boundaries in the independent testing dataset (Syn_D2_; Fig. 4*A*). Even with salt and pepper noise, the Swin transformer detects and segments the neurons accurately (Fig. 4*B*).

### Swin transformer performance

We compared the performance of our Swin transformer to a Mask-R CNN-based algorithm (ANMAF; Tong et al., 2021) and a CNN-based approach (Cellpose; Stringer et al., 2021; Pachitariu and Stringer, 2022). We used GCaMP6s-expressing neurons imaged at baseline (aCSF perfusion) and NMDA perfusion/washout as conditions representing low and high fluorescence, respectively (*n* = 5 brain slices in each condition). We evaluated the algorithms in the independent test dataset (D_2_, *n* = 15 Z-stack images, 8 brain slices) and the independent synthetic test dataset (Syn_D2_).

**Figure 5.**
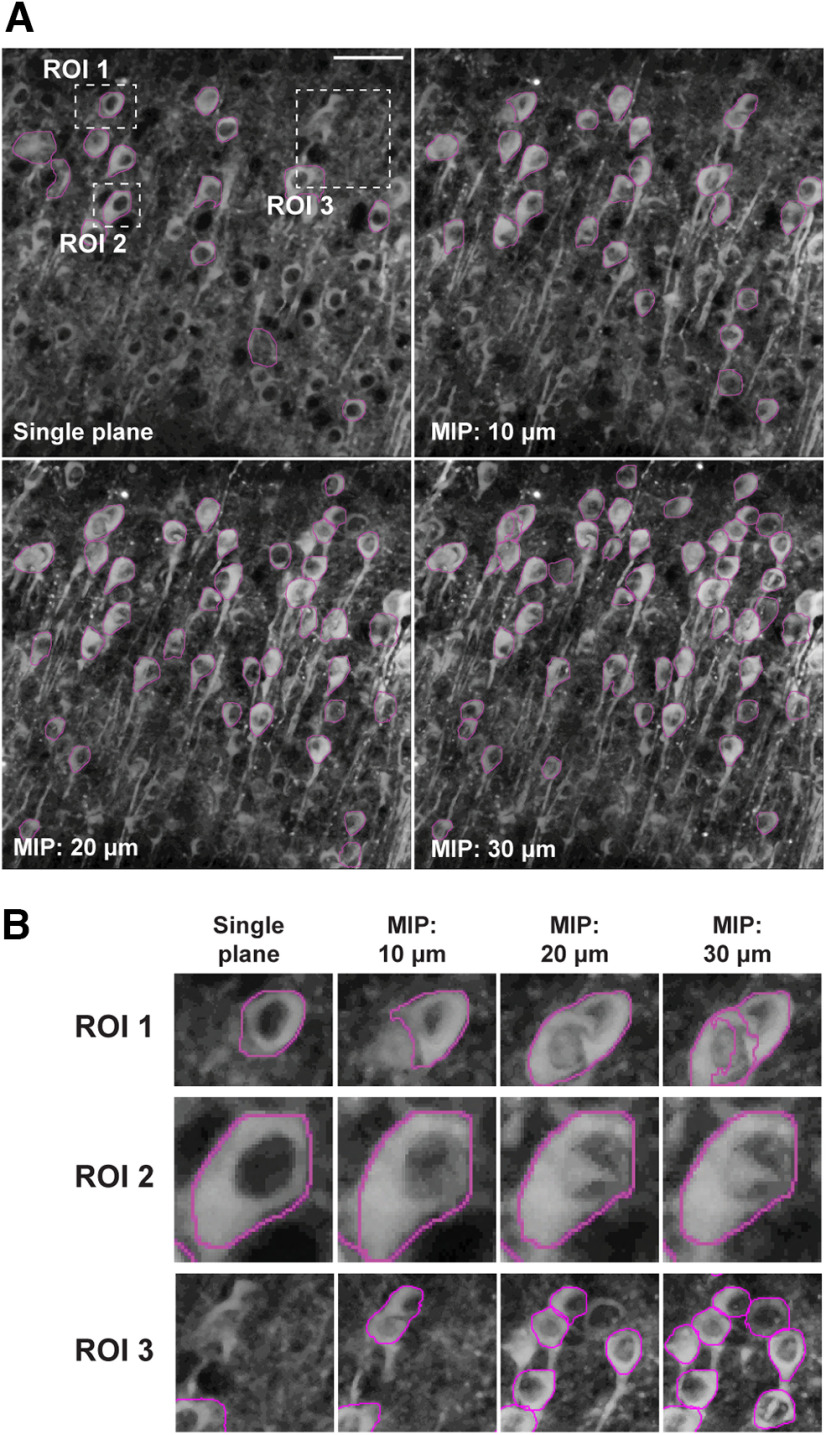
Neuronal segmentation using maximal intensity projections of different depths. ***A***, Maximal intensity projections (MIP) two-photon images z-stacks images of GCaMP6s expressing neocortical neurons imaged at 2-μm steps. Segmented neurons (magenta) are overlayed on each image. ***B***, Three different ROIs from each image in panel A. Notice that the probability of false detection and doublets increases with overlapping neurons (ROI 1), the accuracy of correct predictions (ROI 2), and the total number of segmented neurons (ROI 3) increases with thicker MIPs. Images contrast-enhanced (CLAHE, ImageJ). Scale bar = 50 μm.

We first demonstrated the generality of the Swin transformer to segment maximum intensity projection (MIPs) images at different depths and in single-plane images. The model trained on 20 μm MIP images segments neurons using MIP images of different depths, in addition to single plane images (Fig. 5). The probability of false positives/doublets was higher with the increase in the depths of the MIPs images Region of interest (ROI) 1; however, the accuracy of the predicted boundary (ROI 2), and the total number of detected neurons (ROI 3) was also higher with increased depths. We decided to use 20 μm depth MIPs as it balances accuracy with many neuronal detections, but this depth can be adjusted based on the researcher’s preference.

**Figure 6.**
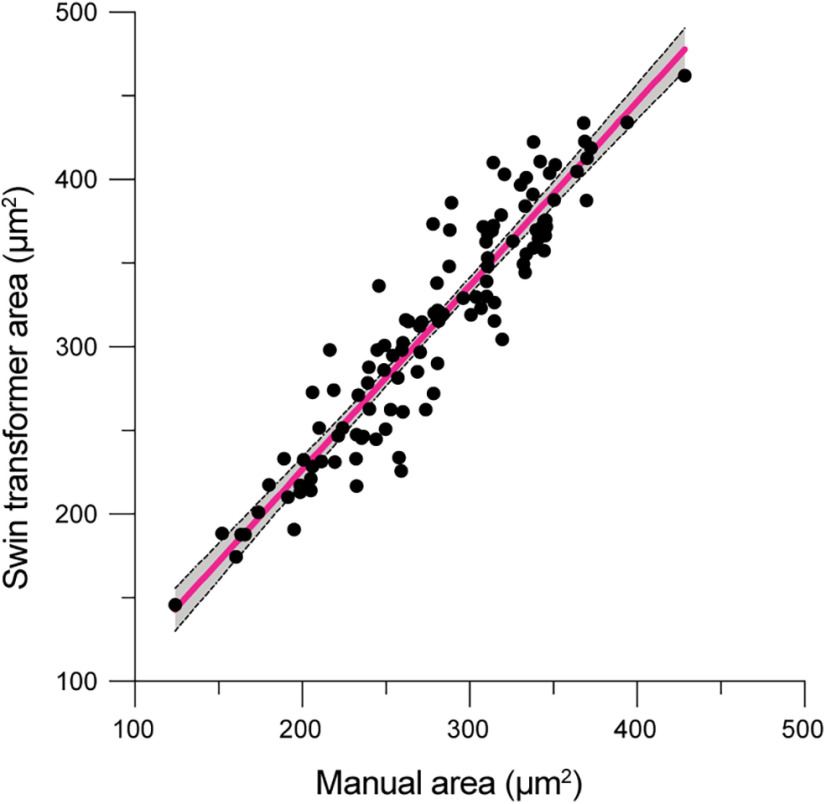
Neuronal somatic area comparison between the Swin transformer and manually detected ones. The mean difference is 34.2 ± 25.6 μm^2^ (SD). Slope 1.10 [95% CI 1.02–1.18]. Pearson correlation *R*^2^ = 0.875; *p* < 0.001. *N* = 113 somatic areas. Line: fit. Dashed lines: 95% confidence intervals.

We then determined the reliability of the Swin transformer for boundary detection by comparing the identified areas with the manually annotated area measurements. The average difference between the Swin transformer and manual areas was 34.2 ± 25.6 μm^2^ (mean ± SD, *n* = 113; Fig. 6). This overestimation of the Swin transformer (12.5 ± 9.3%) could be explained by human annotators consciously avoiding proximal dendrite segments during manual tracing compared with the automated algorithm.

**Figure 7.**
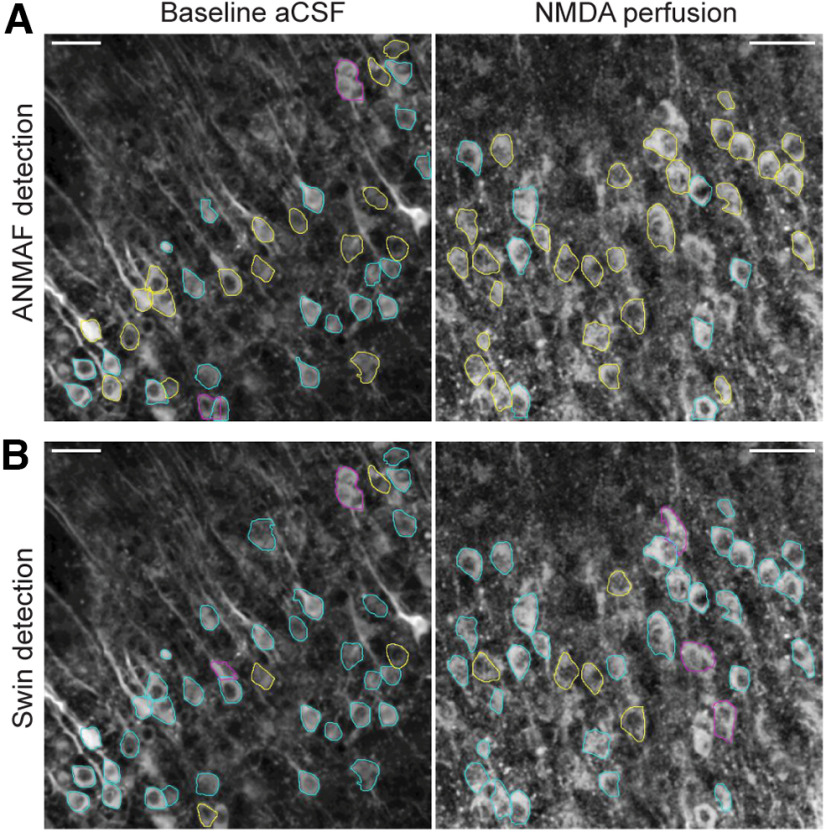
Improved efficacy of neuronal somatic boundary detection by the Swin transformer compared with a Mask-R CNN algorithm. Representative images of GCaMP6s-expressing neurons under baseline aCSF perfusion (left column) and NMDA perfusion (right column). ***A***, ANMAF detected neurons. ***B***, Swin transformer detected neurons. The correct detections are in cyan, missed detections in yellow, and false positive detections in magenta. Images contrast-enhanced (CLAHE, ImageJ). Scale bar = 50 μm.

Next, we evaluated the effect of the detection threshold on the performance of the Swing transformer and compared it to ANMAF. When ANMAF was used to detect GCaMP6s-expressing neurons, whose brightness depends on intracellular Ca^2+^, it failed to detect and segment many neurons compared with the Swin transformer (Fig. 7; Table 2). Next, we varied the Swin detection threshold from 0.05 to 0.4 and analyzed its performance by examining six images (three from baseline and three from NMDA). A threshold higher than 0.05 resulted in a higher percentage of correct detections and a lower percentage of incorrect detections. However, some true positives were also removed if the threshold value was too high, leading to fewer detections. In all conditions, all threshold values achieved a higher percentage of correct detections and a higher number of detections than ANMAF (Table 2). When comparing the Swin transformer performance, we used a 0.05 threshold value to achieve the highest detections (highest recall) and minimize false negatives. This parameter can be adjusted according to the researcher’s preference to achieve high recall (with more false positives) or high precision (with fewer false positives).

**Table 2:** Threshold parameter changes on the Swin transformer performance

	Baseline				NMDA			
Threshold	Total detected neurons	Correct detections	Wrong boundary	FP background and doublets	Total detected neurons	Correct detections	Wrong boundary	FP background and doublets
ANMAF	54	55.6%	38.9%	5.6%	82	43.9%	47.6%	8. 5%
Swin 0.05	105	66.7%	18.1%	15.2%	179	51.4%	26.8%	21.8%
Swin 0.10	98	76.5%	14.3%	9.2%	172	53.5%	26.2%	20.4%
Swin 0.20	92	77.2%	13.0%	9.8%	155	55.5%	25.8%	18.7%
Swin 0.40	82	81.7%	9.8%	8.5%	110	61.8%	21.8%	16.4%

*N* = 3 Z-stack images for the baseline and 3 for the NMDA condition. FP: false positives.

**Figure 8.**
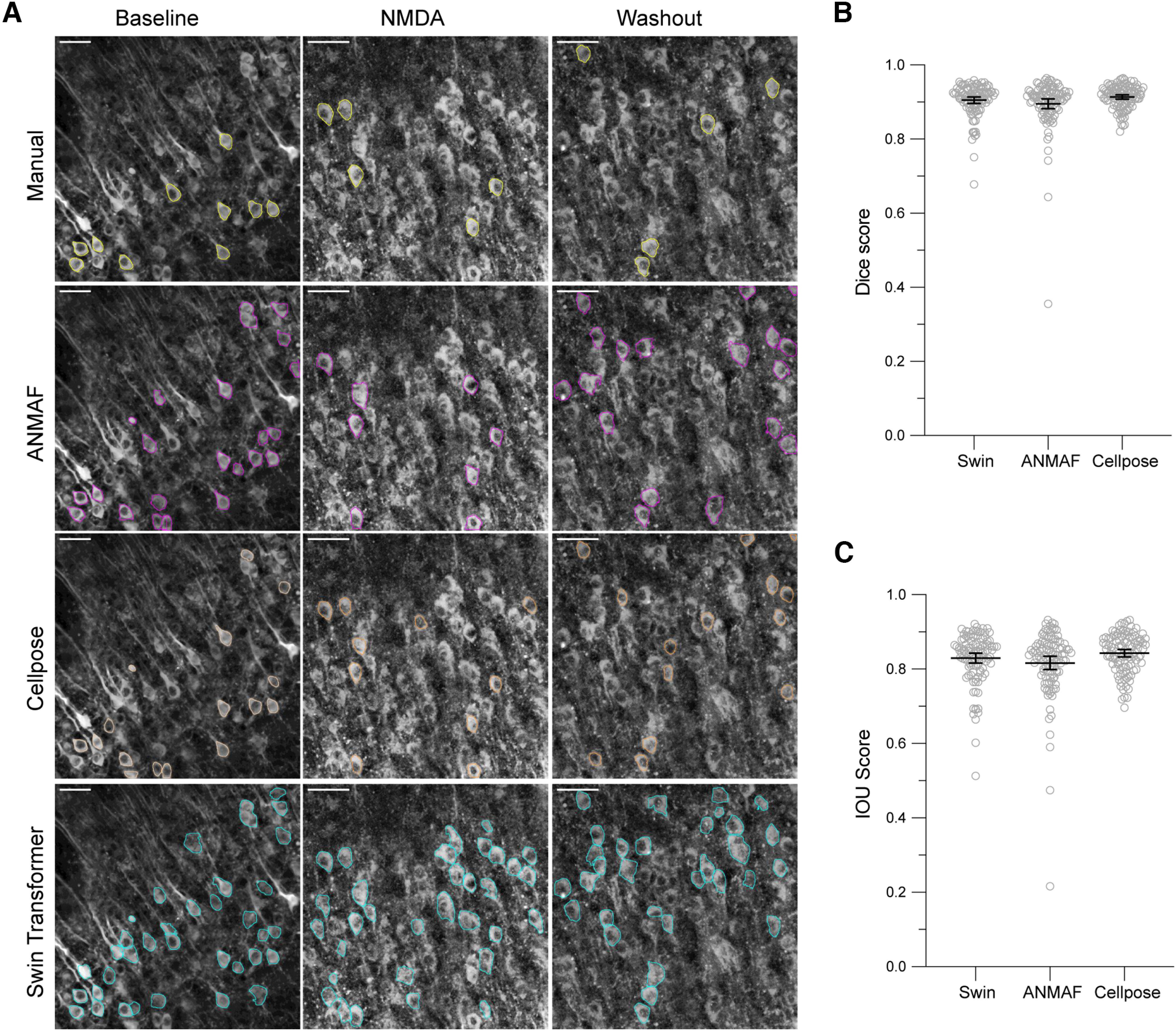
Somatic detected boundaries by Swin transformer, Cellpose, and ANMAF show no difference. ***A***, Left column, Baseline aCSF perfusion. Middle column, NMDA perfusion. Right column, Washout. Yellow outlines in the top row show the manually annotated neuronal boundaries. The ANMAF and Cellpose outputs are labeled with magenta and orange outlines in the second and third rows. The Swin transformer approach detected contours marked in cyan, third row, using a detection threshold value of 0.05. ***B***, Dice scores, RM One-way ANOVA *F*_(2,212)_ = 4.31, *p* = 0.015. Dunnett’s multiple comparison tests, *p* = 0.224 between Swin and ANMAF and *p* = 0.269 between Swin and Cellpose. ***C***, IOU scores, RM One-way ANOVA *F*_(2,212)_ = 4.57, *p* = 0.011. Dunnett’s multiple comparison tests, *p* = 0.248 between Swin and ANMAF and *p* = 0.209 between Swin and Cellpose. Means ± 95% CI. Images contrast-enhanced (CLAHE, ImageJ). Scale bar = 50 μm.

We next compared the performance of our Swin transformer to both ANMAF (Mask-R CNN-based algorithm) and Cellpose (a CNN-based approach; Fig. 8*A*). Here, the ground truth was represented by 131 neurons derived from the expert annotations. We computed the Sorensen-Dice coefficient from all predicted neurons for each ground truth instance of a neuron. The final identified neuron was the one that overlapped most closely with a ground truth neuron (highest Sorensen-Dice coefficient). An instance was labeled as correct if the ground truth neuron overlapped with a predicted neuron with an intersection-over-union (IOU) >0.5. Otherwise, the instance was categorized as a missed neuron. ANMAF correctly detected 118 of 131 neurons (90.1% yield rate). Cellpose also detected 118 of 131 neurons (90.1% yield rate). Instead, the Swin transformer had a higher detection yield of 98.5% (129 of 131 neurons, 0.05 threshold value; Table 3). There was a statistical difference in the Dice and IOU scores between the different algorithms. However, the Swin transformer’s Dice and IOU scores were not different from ANMAF or Cellpose (Fig. 8*B*,*C*). Therefore, the Swin transformer has a higher detection rate without losing accuracy in boundary demarcation.

**Table 3:** Quantitative comparisons between the ANMAF, Cellpose, and the Swin transformer on the manually annotated neurons from the independent testing dataset (D_2_)

Algorithm	Correct detections	Missed neurons				Total neurons
		Baseline	NMDA	Washout	Total	
ANMAF	118 (90.1%)	8	9	2	13 (9.9%)	131
Cellpose	118 (90.1%)	5	3	5	13 (9.9%)	
Swin Transformer	129 (98.5%)	0	2	0	2 (1.5%)	

*N* = 15 Z-stack images, 8 brain slices.

Since the yield ratios were no different between ANMAF and Cellpose, we compared the performance of the Swin transformer to ANMAF. We computed the number of true positives (TPs), false positives (FPs), and false negatives (FNs) for both approaches. Two human experts were provided with all the Swin transformer and ANMAF detections, who classified them as TP or FP. Additionally, they manually contoured the regions of the neurons missed by the approaches to determine the number of false negatives. ANMAF detected 355 neurons, of which 314 were TP, 41 were FP, and 289 were missed neurons (Table 4). The Swin transformer detected 632 neurons, with 518 TP, 103 FP, and 11 instances labeled as “unknown” since the human experts could not categorize them as true or false positives (Table 4). The precision from ANMAF (0.88) was slightly higher than the Swin transformer approach (0.83). However, the Swin transformer approach had a much higher recall (0.86) than ANMAF (0.52; Table 4). Thus, the Swin transformer performs better than a Mask-R CNN algorithm (ANMAF) when neurons express GCaMP6s, with more detections, fewer false negatives, and more true positives.

**Table 4:** Comparison between the approaches for detecting neurons on the independent testing dataset (D_2_)

Approach	Total detected neurons	True positives (TP)	False positives (FP)	False negatives (FN)	Unknown	Precision	Recall
ANMAF	355	314	41	289	-	0.88	0.52
Swin transformer	632	518	103	85	11	0.83	0.86

*N* = 15 Z-stack images, 8 brain slices.

**Figure 9.**
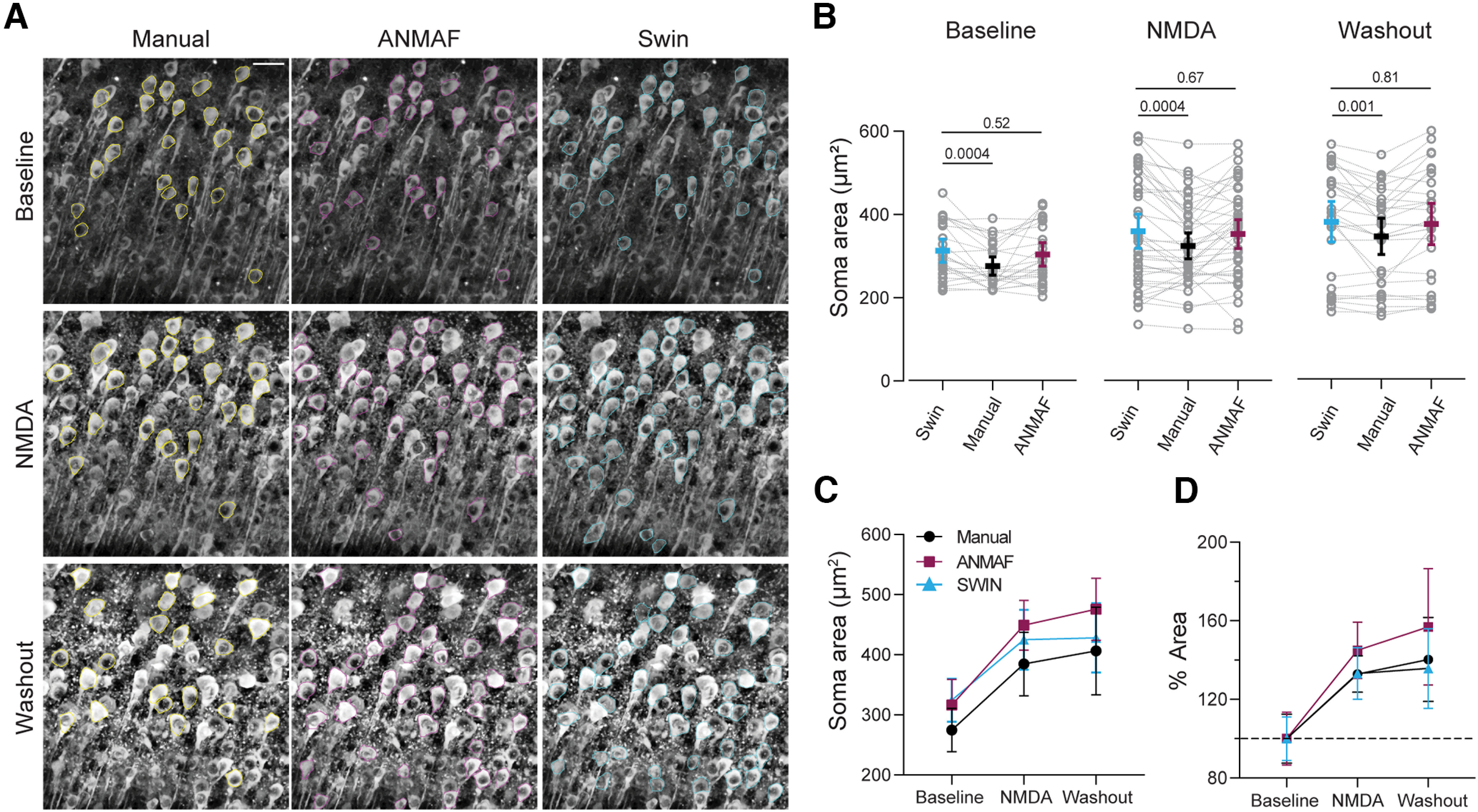
The Swin transformer detects changes in neuronal somatic sizes in the presence of NMDA. ***A***, Representative images showing ROIs obtained using hand tracing (yellow, top), ANMAF (magenta, middle), and Swin transformer (cyan, bottom). ***B***, The Swin transformer calculated higher areas than manually labeled ROIs. Baseline, RM one-way ANOVA, *F*_(2,48)_ = 8.72, *p* = 0.0006, Dunnett’s *post hoc* test; *n* = 25 matched neurons. NMDA: RM one-way ANOVA, *F*_(2,82)_ = 8.695, *p* = 0.0004, Dunnett’s *post hoc* test; *n* = 42 matched neurons. Washout, RM one-way ANOVA, *F*_(2,56)_ = 7.840, *p* = 0.0010, Dunnett’s *post hoc* test; *n* = 29 matched neurons. Two brain slices. ***C***, Matched absolute somatic area change during NMDA-induced long-term neuronal swelling across three detection methods. Two-way ANOVA: Interaction *F*_(4,141)_ = 0.32, *p* = 0.86; Treatment *F*_(2,141)_ = 20.33, *p* < 0.0001; Detection algorithm *F*_(2,141)_ = 2.67, *p* = 0.073. ***D***, Matched area percent change during NMDA-induced long-term neuronal swelling across three detection methods. Two-way ANOVA: Interaction *F*_(4,141)_ = 0.4295, *p* = 0.787; Treatment *F*_(2,141)_ = 22.88, *p* < 0.0001; Detection algorithm *F*_(2,141)_ = 1.381, *p* = 0.255. Manual *n* = 13 matched neurons across treatments, ANMAF *n* = 12 matched neurons across treatments, and Swin *n* = 25 matched neurons across treatments. Two brain slices. Means ± 95% CI. Images contrast-enhanced (CLAHE, ImageJ). Scale bar = 50 μm.

We further evaluated whether the Swin transformer detections capture biologically relevant changes to the neuronal somas during NMDA-induced cell swelling (30 μm NMDA for 10 min) and compared it to ANMAF and manual annotations. All three approaches were used to detect neuronal somatic areas from two-photon images of the same Thy1-GCaMP6s brain slices in three distinct conditions (baseline, NMDA, and washout; Fig. 9*A*). The manual somatic ROIs were hand-traced by one experimenter, and another blinded to the hand-traced ROIs evaluated the automated detections generated by ANMAF and the Swin transformer. Detections labeling the same neurons were paired across different detection methods and conditions based on the ROI spatial location. The Swin transformer neuronal detections had slightly larger areas than manually traced ROIs but not ANMAF-detected neurons (Fig. 9*B*). However, the Swin transformer generated more detections in all three conditions with greater accuracy than ANMAF (Table 5). Importantly, area overestimation by automated algorithms did not affect the estimation of the downstream physiological outcome, as the NMDA-induced prolonged neuronal swelling was captured in all three detection methods to the same extent and not different between detection methods (Fig. 8*C*). Thus, changes to neuronal somatic areas are detected by the Swin transformer as precisely and carefully as hand tracing while eliminating human bias in sample selection. Furthermore, the efficacy of the Swin transformer to detect and segment neuronal somas in NMDA and washout conditions despite high neuropil signal (i.e., low contrast) demonstrates the effectiveness of the Swin transformer in the presence of noise.

**Table 5:** Accuracy of neuronal morphology detections using hand tracing, ANMAF, and the Swin transformer

	Baseline			NMDA			Washout		
Approach	Total identified neurons	Correct detections	Wrong boundary	Total identified neurons	Correct detections	Wrong boundary	Total identified neurons	Correct detections	Wrong boundary
Manual	48	-	-	56	-	-	44	-	-
ANMAF	52	78.9%	21.1%	101	74.3%	25.7%	91	64.8%	35.2%
Swin	70	82.9%	17.1%	140	75.7%	24.3%	114	78.1%	21.9%

*N* = 6 Z-stack images (2 images per condition), 2 brain slices.

**Figure 10.**
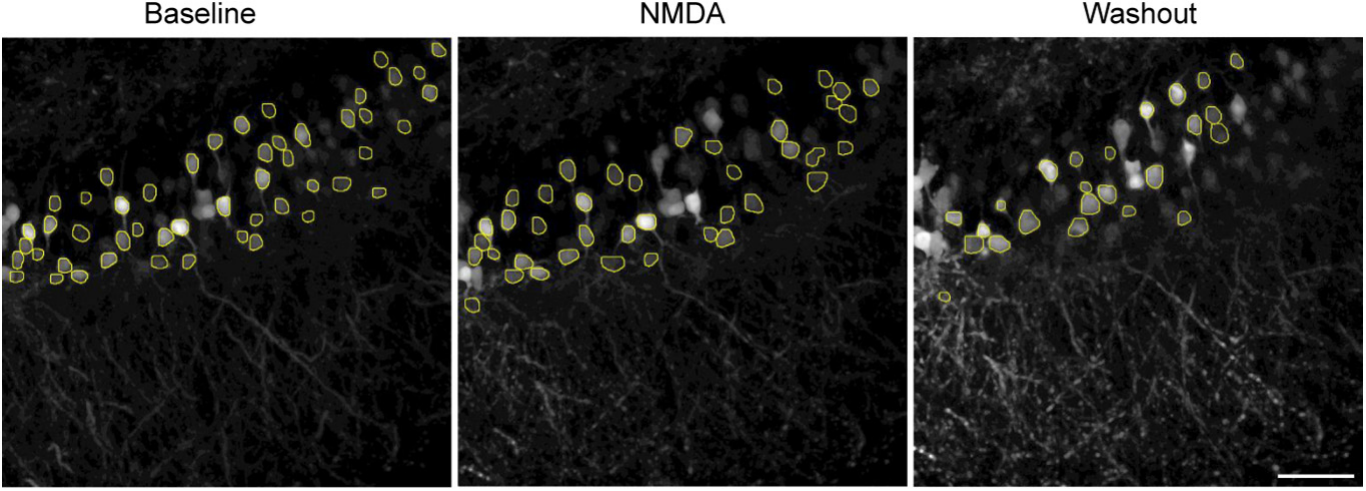
Neuronal boundaries of YFP-expressing neurons in the CA1 region detected by Swin transformer. The Swin transformer was trained with GCaMP6s labeled neurons. Baseline aCSF perfusion (left column) and, NMDA perfusion (middle column), and Washout perfusion (right column). Images contrast-enhanced (CLAHE, ImageJ). Scale bar = 50 μm.

Finally, we evaluated whether the Swin transformer trained with a calcium biosensor (GCaMP6s) can detect neuronal cell bodies labeled with a stable fluorophore and in another brain region with a different cytoarchitecture. We imaged YFP-expressing neurons in the CA1 region of the dorsal hippocampus under two-photon microscopy. Neurons in the pyramidal layer in the hippocampal CA1 region are densely packed compared with the neocortical neurons used to train the Swin transformer. Despite this, the Swin transformer correctly marks cell bodies in YFP-expressing CA1 neurons even when trained with GCaMP6s labeled neurons (Fig. 10).

## Discussion

It is crucial to accurately segment neuronal cell bodies to determine their changes during different physiological and pathologic conditions. Despite the paramount importance, the segmentation of neuronal cell bodies is challenging because of their complex histology. Here, we developed an automated, accurate, reproducible, fast, and unbiased deep-learning-based technique capitalizing on the state-of-the-art Swin transformer (Lin et al., 2021) for neuronal cell body analysis, generalizable to low and high fluorescence conditions. Our algorithm detects and segments 2D somas of GCaMP6s and YFP-expressing neurons from acute brain slices. The Swin transformer excelled in detecting even when trained with a few partially labeled neurons (∼700 manually annotated neurons), eliminating the need for time-consuming human annotations of many cells. It outperformed Mask-R CNN and CNN deep-learning techniques, correctly labeling more neurons. Our Swin transformer has flexibility in its implementation, adjusting to the researcher’s needs. It can detect neurons expressing a different fluorophore if they have a cytoarchitecture similar to the one it was trained for. Its threshold can be adjusted to balance recall and precision, and it can use different MIPs volumes.

Most previous approaches for neuronal cell body segmentation have used fixed, thin tissue slices and imaged them using confocal microscopy or transmitted electron microscopy (Luengo-Sanchez et al., 2015; Falk et al., 2019; Cameron et al., 2020; Hosseini et al., 2020). Also, prior approaches have been evaluated on neuronal cultures or thin brain tissue using a few neurons (Ozcan et al., 2015; Kayasandik and Labate, 2016; K. Xu et al., 2016; Falk et al., 2019; Li et al., 2019; Banerjee et al., 2020; Hosseini et al., 2020). However, thick brain slices (350 μm or more) commonly employed in physiological research have several challenges. These include overlapping neurons from different depth levels, changes in fluorescence brightness throughout the slice, or, in the case of GCamP6s, activity-dependent changes in fluorescence and low contrast between cell bodies and the background, among others. Current deep-learning-based techniques fail to detect and segment the boundaries accurately from thick brain slices, resulting in low recall and Dice scores whenever there is insufficient contrast between the cell and background or when there are many cells. Our Swin transformer approach achieves high detection and segmentation performance in those cases, providing a reliable, automated, fast, and accurate tool for neuronal cell body analysis in thick brain slices. While the Swin transformer overestimated the areas by an average of 12.5% compared with manual tracing, this is systematic and not biased like human boundary labeling, which must decide where to limit the soma versus the dendrite. It is important to note that while we used the Swin transformer to detect neuronal cell bodies using 20-μm maximum intensity projections, it can also be used with different depths of maximum intensity projections and even in a single plane (Fig. 5). For our purposes, we are interested in the maximal area of neuronal cell bodies (Glykys et al., 2019; Takezawa et al., 2023), which is why we use maximum intensity projections. While our work focuses on performing 2D segmentations, extending the approach for 3D segmentations may be possible by replacing the Swin transformer block in Figure 3 with a Swin3D block (Yang et al., 2023). However, this would require exhaustive 3D slice-by-slice annotations of the neurons. Alternatively, the 2D segmentations can be performed on individual slices, and the predictions from the neighboring slices can be used to refine and generate 3D segmentation of neuronal volumes. Yet, this approach requires correlating neuronal boundaries between slices, which, if done manually, would take a significant amount of time. In the future, we plan to apply the current 2D model to segment the neurons on individual slices and then incorporate the information from the neighboring slices. This approach needs to be refined to yield accurate 3D segmentations.

The Swin transformer trained on GCaMP6s expressing neocortical neurons detected two-photon images of YFP-expressing pyramidal neurons in the CA1 region of the dorsal hippocampus. As such, this algorithm should be able to segment neurons labeled with other cytosolic fluorophores in other brain regions if neurons have a fluorescent cytoarchitecture similar to the trained data set. In case the current model does not perform to a user’s standard on a new fluorophore or brain region, more robust segmentation results can be obtained by using the current model as pretrained weights to train a new model using only a few hundred manually annotated neurons of the target fluorophore, or brain region (transfer learning). Importantly, the Swin transformer detects meaningful increases in somatic neuronal areas similar to careful hand tracing, with no bias and quickly.

The Swin transformer approach has some limitations. It has a lower precision than ANMAF (0.83 vs 0.88) because of higher predicted false positives. Some false positives are caused by poor contrast of the fluorescently labeled neurons with the background. Second, the Swin transformer measures neuronal somatic areas using maximum-intensity projections, not neuronal volume (3D segmentation). Third, the Swin transformer detects some doublets, where two adjacent neurons are identified as one instance. Human experts can avoid doublets by examining adjacent slices to obtain information about neuronal boundaries. In the case of the Swin transformer, the edges are predicted from only one image instead of using data from adjacent slices. We plan to address these limitations by developing a semi-supervised approach in the future to measure 3D neuronal volumes, which incorporates the prediction information from neighboring slices (M. Xu et al., 2021; Wang et al., 2022).

To conclude, we developed an automated deep-learning network based on a Swin transformer for 2D neuronal cell body analysis in thick brain slices. This tool can substantially impact neuroscience by detecting and accurately labeling numerous fluorescent neurons, even with a low-emitting fluorophore, leading to more comprehensive and quantifiable studies of neuronal cell bodies. With the help of our flexible algorithm, researchers can study the changes in neuronal somas under various physiological and pathologic conditions using large numbers of neurons without manually annotating them.

10.1523/ENEURO.0148-23.2023.ext1 Extended Data 1 Swin Transformer code. Download Extended Data 1, ZIP file.

## Synthesis

Reviewing Editor: Viji Santhakumar, University of California Riverside Decisions are customarily a result of the Reviewing Editor and the peer reviewers coming together and discussing their recommendations until a consensus is reached. When revisions are invited, a fact-based synthesis statement explaining their decision and outlining what is needed to prepare a revision will be listed below. The following reviewer(s) agreed to reveal their identity: Kate O’Neill.

The manuscript provides a framework for using a Swin transformer to perform fast and automated segmentation of neuron cell bodies in max-intensity images labeled with fluorescence proteins, such as GCaMP6s and YFP. In this revised version, the clarity of the manuscript has greatly improved. Some remaining concerns regarding the generality of this method need to be addressed as this will determine how useful this method will be for the neuroscience community.

1) It is interesting to see that the swin transformer trained on GCaMP6s cortex data can be used to segment YFP signals in the hippocampal CA1. How much does this generalize to other brain regions and with other biosensors? Do users need to train their own transformers for their specified brain region and biosensor? Or will this method generally work well for segmenting neuronal somas with any type of cytosolic staining? It will be helpful if the authors could demonstrate or discuss these points in the main text and discussion section.

2) The authors indicate that this method can work on both max-intensity projection images of different depth and single plane images, but only images with 20μm max-intensity projection are shown. The authors need to provide examples of segmenting max-intensity images with different thickness (10μm, 30μm, etc.), as well as single plane images.

3) The authors need to explain why this method currently does not work on 3D images. Additionally, it will help to discuss potential solutions.

4) Please consider including comparisons with cellpose in Table 3 and Table 4.

5) The authors need to explain why a threshold of 0.05 is chosen in Table 1, as the authors showed that the swin transformer has a lower precision compared to ANMAF.

## Author Response

Dear reviewers,

We want to thank the reviewers for their continued interest, thoughtful comments, and suggestions. The manuscript has been revised based on the reviewers’ suggestions. Changes are marked in the manuscript. We hope that the revised manuscript addresses all of your concerns and that you find it favorable.

Respectfully,

The authors.

Comment 1: It is interesting to see that the swin transformer trained on GCaMP6s cortex data can be used to segment YFP signals in the hippocampal CA1. How much does this generalize to other brain regions and with other biosensors? Do users need to train their own transformers for their specified brain region and biosensor? Or will this method generally work well for segmenting neuronal somas with any type of cytosolic staining? It will be helpful if the authors could demonstrate or discuss these points in the main text and discussion section.

Our Response:

The reviewer brought up an important point to clarify the generality of our algorithm. We discuss this in the Discussion section. We have demonstrated that the Swin transformer trained on synthetic data generated from approximately 700 manually annotated neocortical neurons expressing GCaMP6s can segment YFP-expressing CA1 hippocampal neurons. As such, this algorithm should be able to segment neurons labeled with other cytosolic fluorophores in other brain regions if neurons have a fluorescent cytoarchitecture similar to the trained data set. In case one finds the current model not performing to their standards on a new fluorophore or brain region, more robust segmentation results can be obtained by using the current model as pre-trained weights to train a new model using only a few hundred manually annotated neurons of the target fluorophore, or brain region (transfer learning). We have added this to the discussion. (Discussion): lines 375-381.

Comment 2: The authors indicate that this method can work on both max-intensity projection images of different depth and single plane images, but only images with 20μm max-intensity projection are shown. The authors need to provide examples of segmenting max-intensity images with different thickness (10μm, 30μm, etc.), as well as single plane images.

Our Response:

We thank the reviewer for bringing up this point to show the effectiveness of the Swin transformer for projection images at different depths. We have added a new Fig. 5 demonstrating that the model trained on 20 μm MIP images can segment MIP images of different depths (10μm, 30μm) in addition to single plane images. The probability of false positives/doublets was higher with the increase in the depths of the MIP images (ROI 1). However, the accuracy of the predicted boundary (ROI 2) and the total number of detected neurons (ROI 3) was higher with increased depths. The MIP depth can be adjusted by the researcher based on their preference. Manuscript lines (Result): lines 240-247.

Comment 3: The authors need to explain why this method currently does not work on 3D images. Additionally, it will help to discuss potential solutions.

Our Response:

The scope of this paper is to segment the 2D maximum-intensity projection images, clarified in abstract, significance, introduction, and discussion. It is possible to extend the approach for 3D segmentation by replacing the 2D Swin transformer block with, e.g., Swin3D block (Yang et al. 2023). However, this would require 3D annotated data, with exhaustive slice-by-slice annotations of the neurons. Alternatively, in our future work, we plan to apply the current 2D model to segment the neurons on individual slices and then incorporate the information from the neighboring slices to refine the predictions and obtain the 3D segmentations. We have added this limitation to the discussion. (Discussion): lines 362-372, 392-393.

Comment 4: Please consider including comparisons with cellpose in Table 3 and Table 4.

Our Response:

Both ANMAF and Cellpose use CNN-based approaches. Since the yield ratios between Cellpose and ANMAF were similar (Table 2), in the analyses that followed (Tables 3 and 4), we decided to compare the Swin transformer only with ANMAF, which we already had full access to its performance data. The inclusion of Cellpose in Table 3 and Table 4 would require manual detections of true positive/false positive for each instance of Cellpose predictions, which is time-consuming. Considering that the main objective of this paper is to propose a vision transformer approach for neuronal cell body segmentation, in contrast to the current CNN-based algorithms and that ANMAF is indeed a good representative benchmark for CNN-based approaches, the additional benefit of including Cellpose in Tables 3 and 4 would not be enough to justify the additional needs for time-intensive manual annotations and analysis.

Comment 5: The authors need to explain why a threshold of 0.05 is chosen in Table 1, as the authors showed that the swin transformer has a lower precision compared to ANMAF.

Our Response:

In Table 1, we showed that a threshold higher than 0.05 results in a higher percentage of correct detections, with fewer number of total detections. Hence, the yield rate also decreases with the higher threshold. For comparison with ANMAF, we wanted to compare the threshold value with the highest yield rate; hence, 0.05 was selected. During implementation, this is provided as a variable parameter to be selected by the user according to their application and preference. They may choose a lower threshold to obtain a greater number of detections (high recall) or a higher threshold to obtain a fewer number of detections with fewer false positives (high precision). (Results): lines 263-267, Discussion lines 335-339.
